# Magnetic Supraparticles as Identifiers in Single‐Layer Lithium‐Ion Battery Pouch Cells

**DOI:** 10.1002/cssc.202401142

**Published:** 2024-11-10

**Authors:** Sara Li Deuso, Simon Ziegler, Daniel Weber, Felix Breuer, Daniel Haddad, Stephan Müssig, Andreas Flegler, Guinevere A. Giffin, Karl Mandel

**Affiliations:** ^1^ Department of Chemistry and Pharmacy Friedrich-Alexander University Erlangen-Nürnberg (FAU) Egerlandstraße 1 91058 Erlangen Germany; ^2^ Fraunhofer R&D Center Electromobility Fraunhofer Insitute for Silicate Research (ISC) Neunerplatz 2 97082 Würzburg Germany; ^3^ Development Center X-ray Technology (EZRT) Fraunhofer Institute for Integrated Circuits (IIS) Am Hubland 97074 Würzburg Germany

**Keywords:** Battery recycling, Digital product passport, Identification, Supraparticles, Magnetic particle spectroscopy

## Abstract

The development of effective recycling technologies is essential for the recovery and reuse of the raw materials required for lithium‐ion batteries (LIBs). Future recycling processes depend on accessible information, necessitating the implementation of a digital battery passport. The European battery regulation mandates the use of a machine‐readable identifier physically attached to the batteries for accessing digital information. Since externally applied optical labels are vulnerable to mechanical damage, technologies for identification without these restrictions could be beneficial. This study demonstrates that magnetic supraparticles (SPs) can be used for contactless identification of lithium nickel manganese cobalt oxide (NMC) battery pouch cells *via* magnetic particle spectroscopy (MPS) and that multiple pouch cells can be discriminated based on their specific magnetic code. A comparison of three independent model scenarios revealed that the detection of SPs and the impact on cell performance are dependent on the integration location. The results validate the concept of magnetic identification in metallic environments with MPS as an alternative to optical labeling methods. This study provides a foundation for the development of a new selective labeling and identification technology for batteries, with the potential to facilitate recycling and contribute to a more sustainable future.

## Graphical abstract

As an alternative to externally applied optical identifiers, magnetic supraparticles (SPs) can be used for contactless identification of lithium‐ion battery pouch cells *via* magnetic particle spectroscopy. This study validated the integration and detection of magnetic markers in three model scenarios. Experimental investigation revealed that cell performance and the detection of SPs are highly dependent on the integration location.

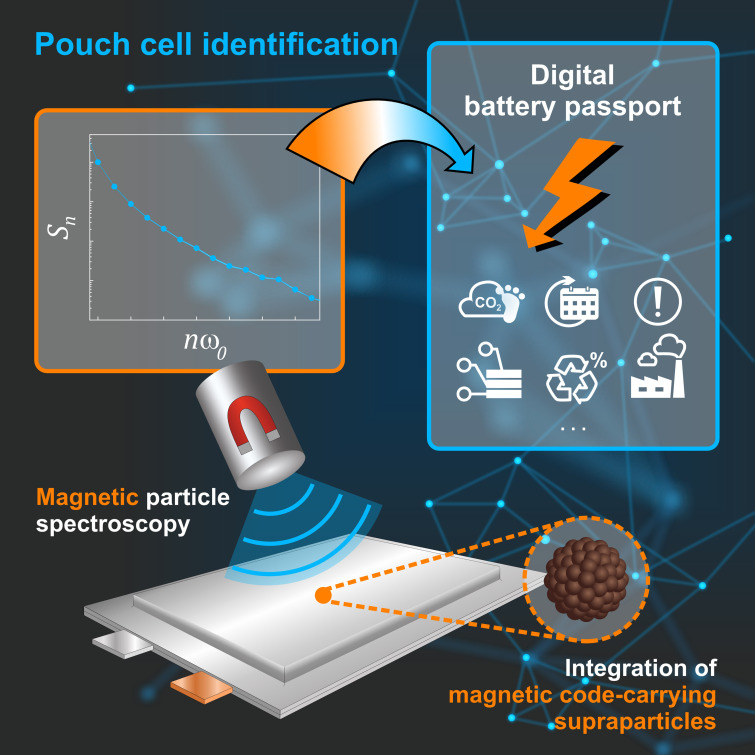

## Introduction

The scarcity of the raw materials,^[1]^ which are required for lithium‐ion batteries (LIBs), necessitates suitable recycling technologies to enable recovery battery materials and their use as secondary sources.^[2]^ Current recycling processes which are economically viable focus primarily on the high value metals. To comply with the EU battery regulations improvements in recycling efficiency are required.^[4]^ However, this comes at the cost of increased process complexity. To address this challenge, design for recycling strategies can be implemented that target automated disassembly of battery cells, material separation and recovery. Efficient and economically viable future recycling processes will rely on the availability of information, particularly about cell chemistry and other parameters.^[5]^

**Figure 1:**
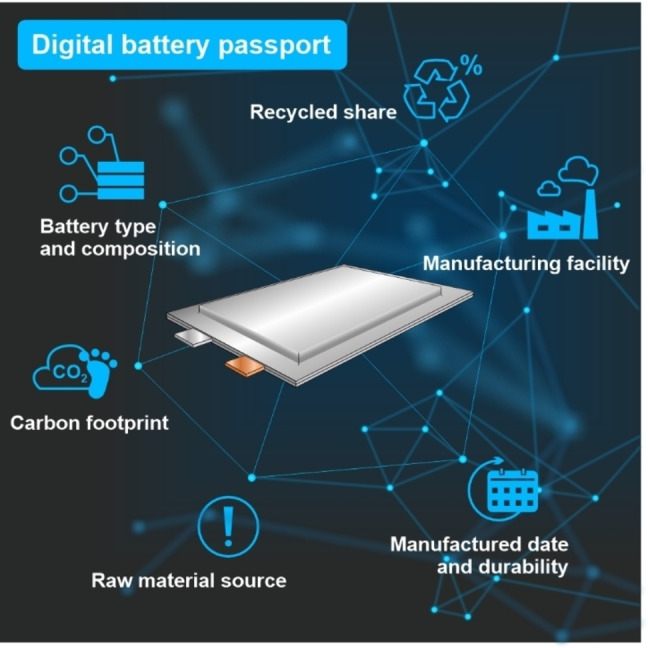
Scheme of exemplary digital battery passport with relevant information about batteries. The battery passport could be physically connected to the battery by an identifier such as a QR code enabling access to tailored product data for suppliers, customers as well as recycling companies.

In linear economy, information is mostly lost when products arrive at the customer′s site due to restricted information transfer.^[6]^ To monitor the ecological footprint, the supply chain and to provide information on the materials used, the establishment of a digital battery passport is required and has been politically demanded (Figure 1). To access data in the battery passport, a machine‐readable identifier must be physically connected to batteries. Externally applied barcodes, serial numbers or QR codes provide an inexpensive and standardized solution.^[4]^ However, these methods are vulnerable to mechanical damage and do not always provide reliable identification. Technologies for identification without such restrictions could be beneficial.

Magnetic supraparticles(SPs), *i. e*. microparticles assembled from nanoparticles (NPs) were recently developed for the identification of objects based on their magnetic properties, resolved by magnetic particle spectroscopy (MPS).^[7]^ The choice of suitable NPs and the arrangement in a defined structure during production determines the magnetic properties and results in a wide variety of SPs with distinguishable signals for identification of arbitrary objects.^[8]^

The signals detected by MPS are associated with the characteristic non‐linear response of the superparamagnetic SPs to an external excitation field. MPS is a fast, sensitive and–above all–non‐invasive measurement technique,^[9]^ to conduct spectroscopic studies on magnetic NPs, most commonly used in biological and biomedical assays.^[12]^ The potential of utilizing MPS to detect different particle‐based, magnetic identifiers as well as recording changes in their magnetic properties caused by environmental triggers like temperature could recently be exploited.^[16]^

The identification of batteries based on the magnetic signal pathway of such micro‐scaled tags could be particularly beneficial because it offers the opportunity to incorporate the identifiers into the casing of the battery cells while ensuring contactless detection from within the unopened LIB cell *via* MPS.^[18]^ Identification of battery cells without the necessity of opening them reduces the likelihood of potential harm during the identification process from electrical or chemical hazards.^[19]^ It has not yet been demonstrated if the magnetic signal can be detected and whether or not the surrounding metallic materials such as the aluminum in the pouch foil, copper foil of anode, aluminum foil of cathode or metal ions within the battery materials affect the magnetism‐based signal response. Furthermore, and in contrast to optical readout methods, it should be possible to read out properties and detect the signal response even in damaged or no longer intact batteries.

**Figure 2:**
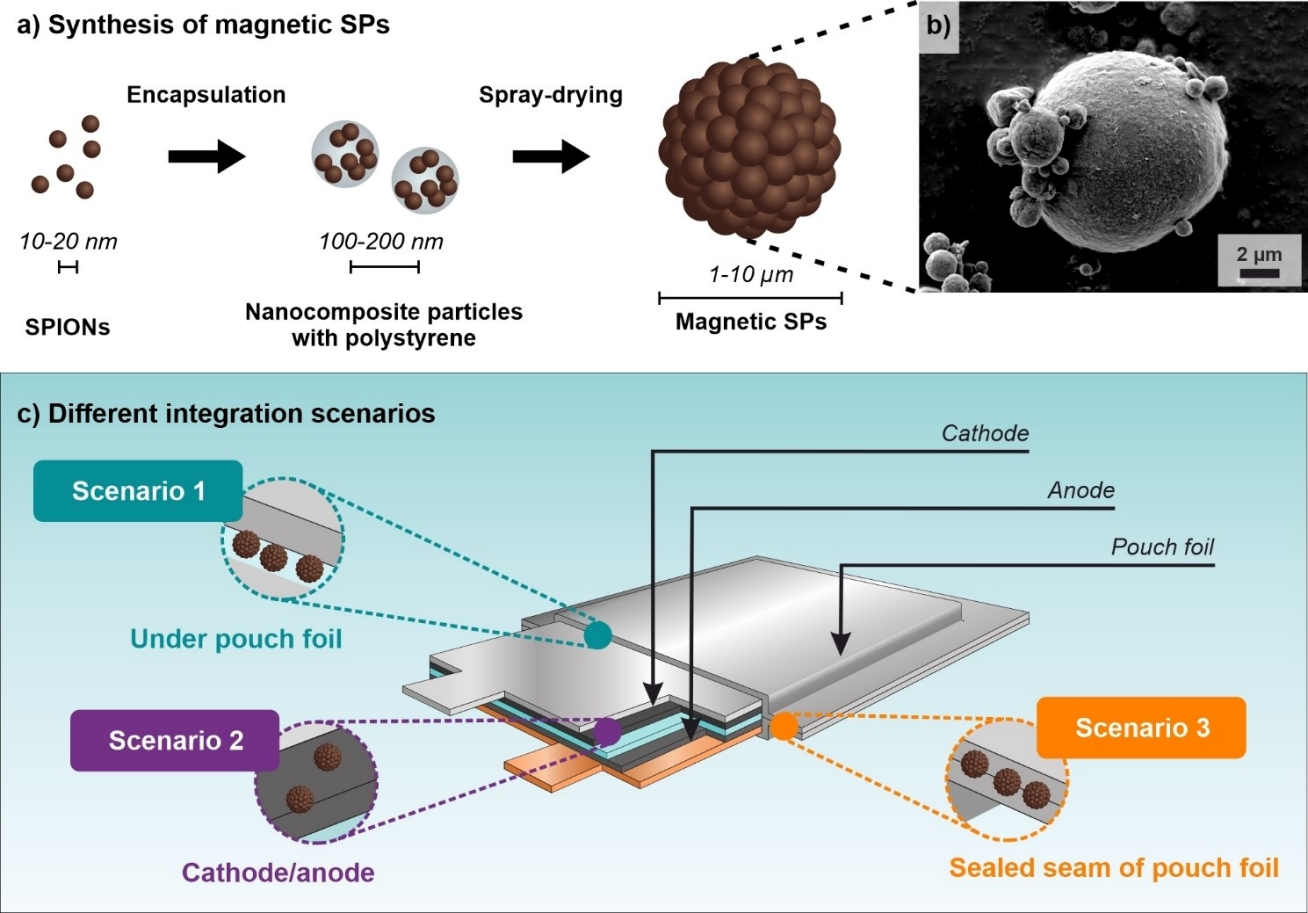
SPIONs are combined with polystyrene to nanocomposite particles and subsequently spray‐dried to synthesize magnetic code‐carrying SPs (a), depicted by a SEM micrograph (b). The scheme shows the selected integration scenarios (c) of magnetic SPs fixed under the pouch foil (scenario 1, turquoise), mixed with active material of cathode and anode (scenario 2, violet) and sealed in seam of pouch foil (scenario 3, orange).

In this work, it is shown that magnetic SPs can be used for contactless identification of lithium‐ion battery pouch cells. The integration and detection of SPs is experimentally validated in three independent model scenarios. In scenario 1, SPs are fixed underneath the pouch foil (Figure 2c). In scenario 2, SPs are integrated as additive in the electrode slurry and homogeneously distributed in the electrode. In scenario 3, SPs are fixed in the sealing seam of the pouch foil. A handheld sensor with flexible, modular sample positioning option is used for signal acquisition based on the principle of magnetic particle spectroscopy (MPS). The aim of this work is to give an overview of the advantages and potential challenges of each scenario in terms of technical feasibility, electrochemical performance, and processing aspects rather than optimizing the process and material efficiency. Finally, magnetic SPs with different magnetic properties are used to identify and distinguish multiple differently marked pouch cells.

## Results and Discussion

### Magnetic Supraparticles for Pouch Cell Identification

For identification of pouch cells, multi‐hierarchical magnetic SPs are used as identifiers. In a first step, superparamagnetic iron oxide nanoparticles (SPIONs) are synthesized *via* a co‐precipitation reaction. The magnetic nanoparticles are subsequently combined with a spacing material, herein polystyrene, to hierarchic sub‐structures, and so‐called nanocomposite particles. Upon forced assembly (spray‐drying) of the nanocomposite particles, micrometer‐sized SPs with magnetic properties as determined by the nanoparticles used and their structural assembly are obtained (Figure 2a and b, more information is provided in the Supplementary Information). To facilitate the evaluation process, the same SP type is integrated in each of the following scenarios. A detailed characterization (MPS, superconducting interference device SQUID measurements, scanning electron microscopy SEM, laser light diffraction) of the employed SPs can be found in the Supplementary Information (Figure S1a–d).

### Integration Scenario 1: Fixed Under Pouch Foil

**Figure 3:**
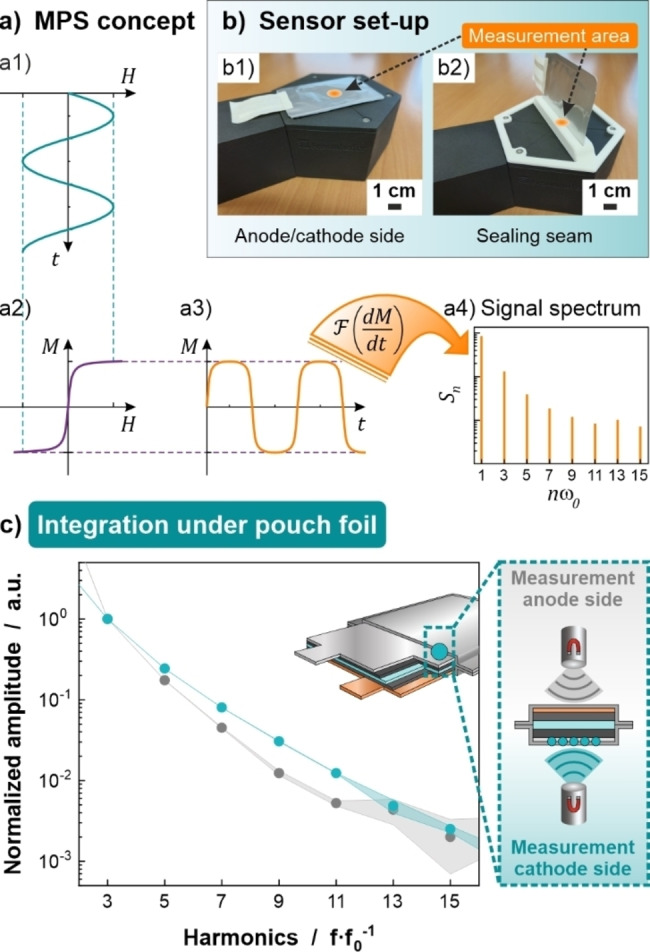
The measurement principle and magnetic signal detected by MPS characterization are shown in (a). Sensor setup upon detection of magnetic identifiers. The orientation of the sensor to the measured cell is varied depending on the integration position (b). For SPs fixed under the pouch foil or integrated in the cathode/anode material, the cell is flatly placed on the sensor and for SPs sealed in the pouch foil seam, an upright alignment of the cell to the sensor is chosen. MPS spectra of SPs integrated under the pouch foil reveal an alteration of the spectral magnetic signal depending on the orientation of the full cell to the sensor (depicted on the right side) during the measurement (c).

In integration scenario 1 (Figure 2c), SPs are located on the inside of the pouch foil on the cathode side of the battery cell (Figure 3). This model location enables the investigation of electromagnetic shielding by surrounding materials but does not intend to demonstrate an industrially feasible integration process. The MPS spectrum is dependent on the field at the location of the SPs, which is in turn influenced by all metallic materials that are in the sensitivity range of the MPS sensor. This implies that the MPS spectrum of raw SPs is not necessarily identical to the MPS spectrum of particles within a battery environment (further explained in the Supplementary Information). The pouch foil of a battery cell is a multilayered foil containing polyethylene terephthalate and nylon as carrier layer, an approx. 30 μm thick aluminum barrier layer as well as polypropylene sealing layer.^[20]^ The magnetic SPs were attached to the inner side of the polymer‐aluminum‐polymer composite film using polyamide adhesion tape. Subsequently, a functional battery cell with a NMC811 cathode and a graphite anode was built with this pouch foil. For the MPS measurements, a complete pouch cell is placed on a MPS sensor (the sensor is in contact with the cathode side of the cell, Figure 3b) and an alternating magnetic field (~20 mT @ 20 kHz) is applied. After the Fourier transformation of the sampled signal response, the MPS spectrum is obtained in the odd harmonics of the excitation frequency (Figure 3a). This spectrum acts as a code for this SP in this specific context. Before the actual measurement, a reference pouch cell without magnetic SPs is measured to calibrate the power amplifiers of the MPS system. This is essential as the pouch cell itself generates a very strong MPS signal, which is eliminated by calibration. The background‐free signal of the magnetic SPs can be detected by subtracting the signal of the calibration signal from the actual measurements.

MPS spectra are shown as the harmonic amplitude as a function of higher harmonics. The spectrum of a number of averaged measurements (measurement time of 1 s for a single measurement) from the cathode side is shown in Figure 3 (turquoise circles) with the respective standard deviation. The averaged MPS spectra of the integrated SPs were normalized to the amplitude intensity of the 3^rd^ harmonic *A_3_* to eliminate the influence of non‐magnetic materials and for better comparison of the measurements throughout this work. It is important for this integration scenario that a comparably good signal‐to‐noise ratio (SNR) is achieved for the measurement through the metal‐containing pouch foil, allowing a reliable signal up to the 11^th^ harmonic to be detected. This decay of the MPS spectrum is used for a reliable identification of the SPs.

When the MPS measurements are carried out from the anode side of the cell where the field must pass through three metallic foils (pouch foil, copper foil of anode and aluminum foil of cathode), the amplitude of the MPS signal is decreased (Figure 3c, gray circles). A weaker signal intensity and therefore a lower SNR compared to measurements from the cathode side limits valid information about the particles up to the 9^th^ harmonic. The reason for the altered response of the magnetic SPs might be related to electromagnetic shielding. In MPS, an electromagnetic field is created by a time‐varying current passing through a coil. If an electrically conductive material, *e. g*. a metallic foil, is brought into this alternating electromagnetic field, an eddy current will be induced in the material, as predicted by Faraday′s Law.^[21]^ The eddy current generated will in turn induce an electromagnetic field which, according to Lenz′s law,^[23]^ counteracts the initial magnetic field. Thus, the particles may only experience a weakened magnetic field strength. It is well known from literature that MPS signal decays are significantly dependent on the applied magnetic field strength.^[24]^ The electromagnetic shielding depends on the number of metallic foils and their thickness which results in different signal responses for different battery cell orientations. This leads to the conclusion that not only the overall (metallic) environment around the magnetic SPs is important for the MPS signal response but also the metallic components between sensor and SPs.

In summary, this scenario is feasible in principle for identification of the magnetic SPs used. Similar MPS results have been obtained for second pouch cells prepared in identical manner (see Supplementary Information Figure S2 for the magnetic characterization of the second cell–for all scenarios). The signals from the two cells indicate the reproducibility of both, the cell preparation (including SP integration) and the magnetic readout. However, the MPS signal response varies depending on the macroscopic orientation of the pouch cells which complicates reliable identification. Strategies to ensure identical orientation of the battery cell to enable a practical application must be developed or alternatively further data processing could be investigated in future work. In addition, a more industrially feasible integration process must be established. It is critical that the SPs do not infer the electrochemical performance of the cell and that a comparably good SNR is obtained in this scenario.

### Integration Scenario 2: Integration in Electrodes

**Figure 4:**
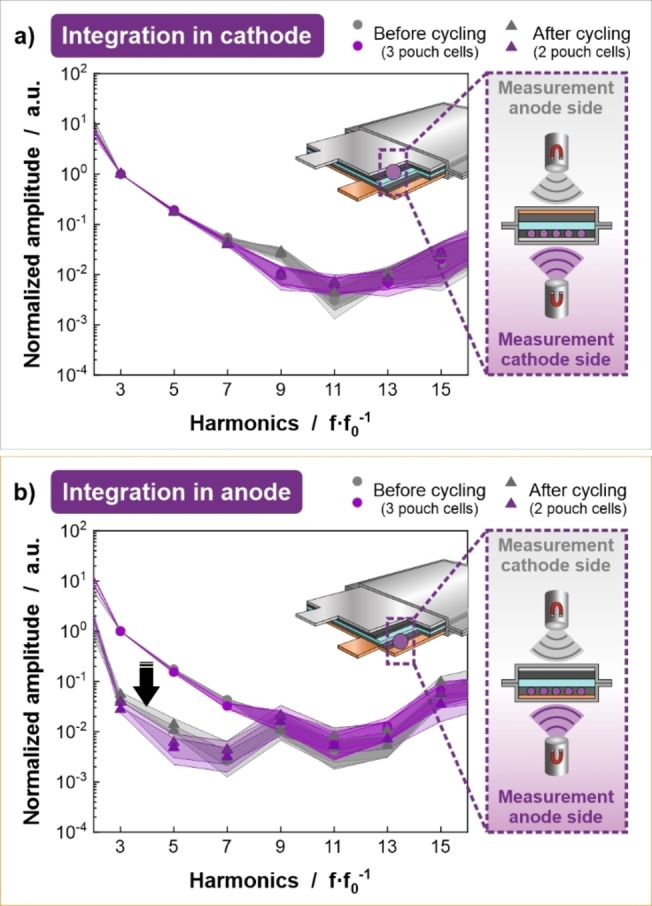
MPS spectra of SPs integrated in the cathode of multiple pouch cells reveal reproducible magnetic signals before (circles) and after cycling (triangles) regardless of the orientation of the full cell to the sensor during the measurement (a). After cycling of full cells with SPs integrated in the anode (b, triangles), the MPS signal intensities are drastically reduced compared to the MPS signal responses of uncycled cells (b, circles). In this case, all MPS spectra were scaled with a common normalization factor to show the effect of the cyclization.

In the second scenario, SPs are distributed in the electrodes to enable detection. This would allow different electrodes to be distinguished to facilitate sorting. Integration of the SPs was tested in either the anode or the cathode to evaluate the feasibility of this integration scenario (the counter electrode was always an electrode without SPs). The integration is performed during the slurry preparation by adding 10 wt % of SP powder in relation to the total powder mass used as an additional last step in the mixing process as described in detail in the Supplementary Information. Adding the SPs during slurry preparation has the advantage that it can be added in the same manner as other electrode additives, thereby minimizing processing effort. However, several major challenges related to the integration process and the electrochemical performance of the electrodes must be solved. The SPs must remain magnetically and mechanically stable during mixing, electrode coating and calendaring. As compared to integration scenario 1, additional metallic foils of cathode (aluminum) and anode (copper) are present between SPs and MPS sensor which may hinder reliable signal readout. Finally, the SPs must not affect the electrochemical performance of the electrode. The magnetic signal response of SPs present in the cathode of a fresh pouch cell, which has not been electrochemically cycled, is slightly shifted to lower amplitudes compared to the signal response obtained for the particle powder fixed underneath the pouch foil. This causes an expedite approach of the detection limit and thus decreased number of resolved harmonics. The high standard deviation around the averaged values could be attributed to lower concentration of SPs in the measured volume upon mixing the powder into the electrodes. A qualitative distinction of the signals before (Figure 4a, violet and gray cycles) and after cycling (Figure 4a, violet and gray triangles) is hardly possible as shown for multiple pouch cells. Thus, the integration process in the cathode does not seem to affect the integrity of the magnetic SPs. MPS measurements can be conducted from either side of the pouch cell. Nevertheless, a reproducible detection of the SPs is only feasible up to the 7^th^ harmonic.

By integrating the magnetic marker particles in the active material of the anode, which has not been electrochemically cycled, the MPS measurements show comparable signal responses as for integrated SPs in the cathode material (Figure 4b, violet and gray circles). However, for cycled cells the spectral magnetic signals rapidly decay (Figure 4b, violet and gray triangles), immediately approaching the detection limit indicating change of the marker particles. XRD measurements before and after cycling confirm a phase transition of magnetite associated with its reduction (Supplementary Information Figure S4d). Therefore, a reliable MPS signal is not detectable.

**Figure 5:**
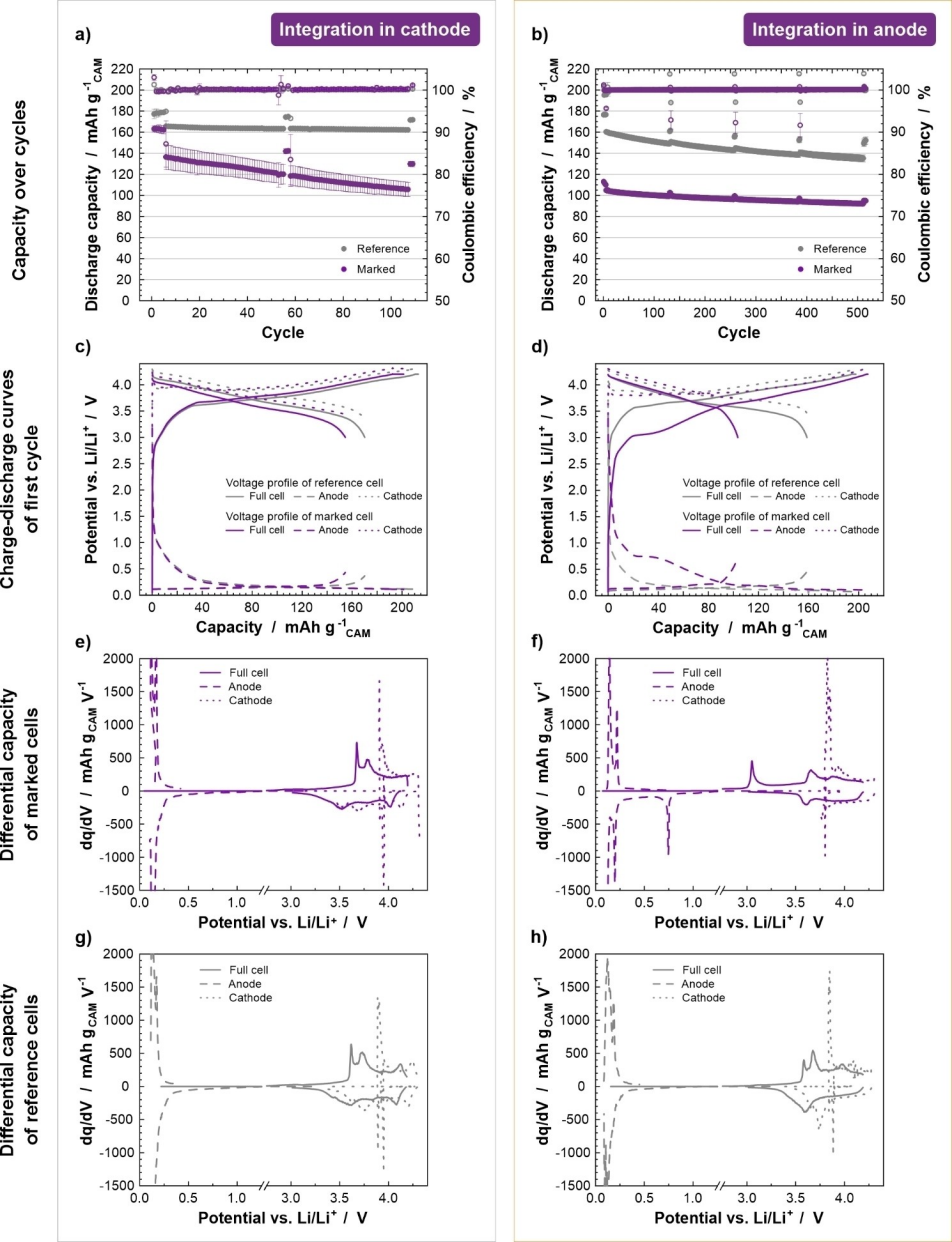
Electrochemical analysis of the full cells with SPs integrated in the electrodes as well as the corresponding references, with (a) and (b) showing the cycling test and (c) and (d) showing the voltage profiles of the first cycle over the capacity for the cathodes and anodes, respectively. (e) and (g) illustrate the differential capacity plots of the marked cathode cells and the reference, while (f) and (g) present the differential capacity plots of the marked anode cells and the reference

The influence of the SPs on the electrochemical performance and vice versa is a critical aspect in this scenario. The performance with SPs integrated in the cathodes was investigated by cycling the full cells at 0.5 C for 100 cycles after a formation step of 5 cycles at 0.1 C. The discharge capacities over the cycles are plotted in Figure 5a compared to reference cells without SPs. The results show an initial decrease in capacity of around 29 mAh g_CAM_^−1^ from 165.68 mAh g_CAM_^−1^ of the references to 136.37 mAh g_CAM_^−1^ of the marked cells and further capacity loss during cycling, resulting in a capacity retention (CR) of 77 % after 100 cycles (capacity of cycle 1 compared to cycle 100 at 0.5 C). In comparison, the reference cells had a capacity retention of 98 %. Further investigation was conducted using electrical impedance spectroscopy, as illustrated in the Nyquist plot in Figure S3a for the cell at 3.8 V. The measured impedance can be divided in two semi‐circles.^[16]^ The high frequency area is attributed to the solid electrolyte interphase, while the low frequency area is attributed to the charge transfer resistance. Compared to the reference cell, the SPs in the cathode cause a major increase in resistance of the high frequency semi‐circle from approximately 0.5 Ω cm^2^ to about 15 Ω cm^2^. A smaller increase occurs in the low frequency semi‐circle from about 1 Ω cm^2^ to approx. 8 Ω cm^2^. In total, the impedance is more than 10 times higher than the reference cell which causes higher polarization and explains the lower discharge capacity. The relatively high amount of 10 wt % of SPs in the cathode might impede the charge transfer pathways within the electrode, as implied by the SEM image in Figure S4a. However, there is no indication that the SPs are electrochemically incompatible with the result of the cathode. Instead, they seem to act as an inactive additive within the electrode. The assumption is supported by evidence presented in Figure 5c, e and g by similar voltage profiles and differential capacity plots of the first cycle compared to the reference cell. The additional reaction peak visible above 4 V in the differential capacity plot of the reference (Figure 5g) is absent in the cells with integrated SPs due to a shift to higher voltages caused by the higher polarization. Furthermore, the SEM images in Figure S4a2 show no differences in the cathode structure after cycling compared to the pristine cathode in Figure S4a1. In addition, equivalent diffractograms before and after cycling are shown in Figure S4c, with only a slight change in the NMC reflection angles due to lithium loss during cycling.

The SPs were also integrated in anodes and tested in full cells with the reference cathodes. Cells were subjected to five formation cycles at 0.1 C before cycling at 1 C for 500 cycles (Figure 5b). A higher C‐rate was possible with the SPs in the anode as the total resistance of the cells was much lower, under 2 Ω cm^2^, and almost equivalent to the reference than when the SPs were present in the cathodes (Figure S3b). Despite the lower cell resistance values, as compared to the cells with SPs in the cathodes, the initial discharge capacity is significantly lower by 56 mAh g_CAM_^−1^ from 160.31 mAh g_CAM_^−1^ to 104.83 mAh g_CAM_^−1^ when the SPs are integrated in the anode despite similar first cycle charge capacities (Figure 5d). This implies that there are irreversible reactions occurring during the first charge cycle. Evidence of such reactions can be seen at around 0.75 V in the voltage profile of the full cell and the anode of the first charge (Figure 5d) and the differential capacity plots in Figure 5f (Anode) compared to the reference cells. This reaction is likely associated with the conversion reaction of iron oxide. Iron oxide has been reported as a conversion anode material and undergoes reduction to Fe and Li_2_O.[^25^, ^26^] The reversibility of this reaction is relatively poor and is associated with a loss of lithium inventory. After the initial cycles, the loss of capacity in the subsequent cycles is comparatively small and the capacity retention after 500 cycles is 88 % (capacity of cycle 1 compared to cycle 500 at 1 C). The reference full cell has lower CR of 84 %, which is attributed to the higher amount of charge transferred during cycling, which likely increases the stress on the electrodes. The structural change within the SPs is also evident in the SEM and XRD data shown in Figure S4b and d. The SEM images highlight a formed layer around and likely in between the SPs related to the mentioned Li_2_O formation (white arrow). The XRD reflection at 32.43° is also assigned on this. The reduction of magnetite is further detectable by the change of its reflections at 30.26°, 35.60°, 43.38°, 57.25° and 63.00°. Similar structural changes were reported for the conversion of iron oxide to Fe and Li_2_O by Yu et al.^[25]^ The integration of SPs in the cathode appears to be a viable approach from the perspective of detection and sorting but challenging due to the impact of the SPs on the electrochemical performance. The negative effects on the cell performance might be reduced by using fewer particles in the cathode, although at the cost of the signal‐to‐noise ratio of the MPS signal. In the case of the anode, protection of the SPs from electrochemical reaction *via* a suitable encapsulation may be a feasible approach. Further research is required to ascertain the viability of this implementation scenario.

### Integration Scenario 3: Fixed in Sealed Seam of Pouch Foil

**Figure 6:**
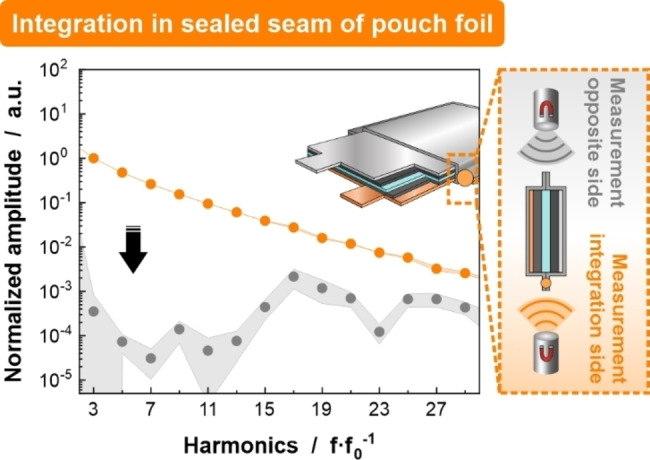
MPS spectra of SPs integrated the sealing seam of the pouch foil show a dependence on the orientation of the measured battery cell. While the detection of the SPs from the side of integration reveals the highest SNR (orange circles), it is demonstrated that SPs cannot be detected from the opposite side (gray circles). In this case, both MPS spectra were scaled with a common normalization factor to represent the absolute signal loss.

Aiming to circumvent electrochemical interference by the used SPs while keeping the processing effort as low as possible, SPs could also be integrated in the sealing seam of the pouch foil. After addition of a few milligrams SP powder between the to‐be‐sealed pouch foil sides, the standard procedure for temperature‐induced sealing was conducted (for more information see Experimental Section in Supplementary Information). In this scenario, SPs must withstand the applied temperature and pressure, while the sealing seam must maintain its functionality. For SPs to be integrated in the sealing seam, the alignment of the cell to the sensor affects the MPS signal response. The detection of SPs from the integration side is feasible. The MPS response of the particles can be resolved up to the 29^th^ harmonic providing the best SNR among all integration scenarios (Figure 6, orange circles). The electromagnetic waves do not penetrate through the thickness of the pouch foil (in the z‐direction) but are rather detected in the x‐direction at the interface of the sealing seam. This enhances the number of detectable higher harmonics before approaching the detection limit. It is likely that this is due to a change in the metallic material surrounding at the SPs location. It is not possible to obtain a reliable signal from the particles when measuring from the opposite side, with the entire pouch cell in between the sensor and SPs (complete disappearance in measurement noise). This is due to the increased material amount and distance between the marker particles and the sensor (Figure 6, gray circles). Therefore, the sensor must be placed next to a sealing seam with integrated SPs either by integrating SPs on all sides or by suitable sensor positioning.

Stability tests were conducted on the sealing seam to ascertain the impact of the presence of SPs in the investigated pouch foil seam. These could potentially pose a safety hazard in the event of a thermal runaway by affecting the seam stability and therefore the maximum pressure and the position of the seam failure were used as a functionality assessment. Consequently, a hose fitting was integrated into a punched hole in the center of a pouch foil which was used to seal an empty full cell pouch bag. The pouch bag was manually inflated with compressed air *via* a precision manometer until a change in the sealing seam or its failure (tearing) could be detected (see Figure S5 in Supplementary Information). Due to the rectangular pouch cell design, the particle integration was tested on both the shorter and the longer pouch cell side. A comparison of the reference and the as‐prepared pouch bags revealed that the stability of the sealing seam was influenced to varying degrees, dependent on the side of integration. The maximum pressure until tearing of the reference pouch bag was 3.1±0.3 bar, consistently on the longer side. The pressure measurements for the sample pouch bags revealed a mean of 2.6±0.2 bar and 1.3±0.3 bar on the short side and the longer side, respectively. The position of seam failure was consistently observed at the integration side for all measurements. In conclusion, this integration scenario provides optimal conditions for both detecting the magnetic SPs and maintaining the cell performance. However, the stability of the sealing seam is slightly reduced upon SPs integration. This effect might be mitigated by reducing the quantity of particulate powder used, while still maintaining optimal detectability.

### Evaluation of Integration Scenarios

**Figure 7:**
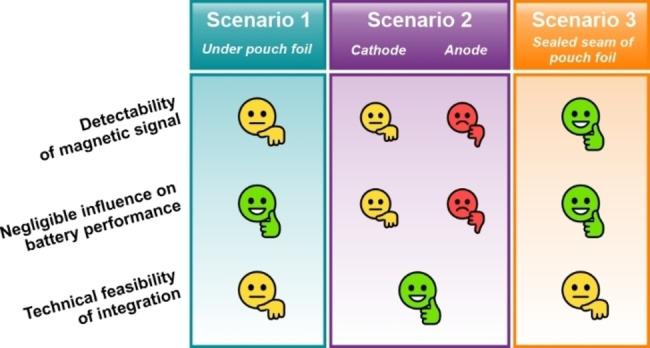
Overview and comparison of detectability, the electrochemical performance, and the technical feasibility of identification of LIB pouch cells with magnetic SPs.

Following the individual testing of the three model scenarios, a comparison and rating of the detectability of the magnetic SPs with MPS, their influence on the performance of the pouch cell, and the practicability of the integration process in industrial battery production was conducted (Figure 7). The highest number of harmonics was detected in the MPS when the SPs were integrated in the sealing seam of the pouch foil. Therefore, scenario 3 provides the most reliable signal outcome presumably due to the positioning of the sensor in the x‐direction at the interface of the sealing seam. When the particulate markers are fixed under the pouch foil (scenario 1), a comparable SNR is obtained when measuring MPS through the metallic pouch foil. It should be noted, however, that the detectability of the SPs is dependent upon the orientation of the sensor in both scenario 1 and scenario 3. In scenario 2, it was possible to utilize SPs integrated in the cathode to identify pouch cells. In contrast, the detection of SPs in the anode was not possible.

Additionally, the integration in both electrodes influences the electrochemical performance to a certain extent, which is crucial for the application. Since the SPs fixed in the sealing seam and under the pouch foil are separated from the electrochemically active part of the battery, they do not affect the performance of the electrodes. However, the integration of particulate markers in the scenarios necessitates the implementation of additional procedures during the fabrication of pouch cells. This requires the development of feasible processes for industrial applications. In contrast, the integration in the electrodes can be performed in a straightforward manner by combining the magnetic additive with the active material powders.

### Pouch Cell Differentiation Based on Different SPs

**Figure 8:**
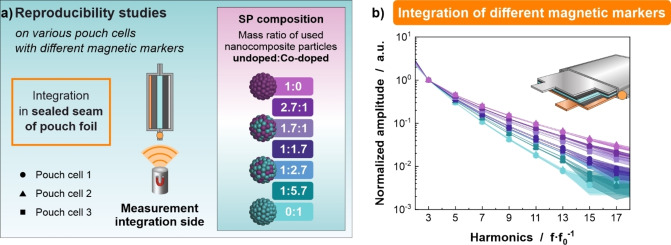
SPs with different compositions were fabricated and integrated in the sealed seam of the pouch foil (a). The signal alteration was achieved by using undoped SPIONs and Cobalt‐doped SPIONs as signal carriers. Nanocomposite particles produced from one of the two components were subsequently combined in different ratios, yielding SPs with only undoped building blocks (1:0, violet hue), various ratios (2.7 : 1, 1.7 : 1, 1 : 1.7, 1 : 2.7, 1 : 5.7) and SPs consisting of only doped nanocomposites (0 : 1, green hue). MPS measurements of pouch cells containing such SPs (three cells per used SP species are shown) reveal faster decaying spectra with decreasing share of the undoped component, thereby facilitating to distinguish different pouch cells (b).

Based on the finding that integration of marker particles into the pouch foil provides the most reliable signal outcome and no electrochemical interference, it was investigated whether pouch cells can be distinguished from different cells when magnetic SPs with different magnetic properties are employed. Therefore, SPs with seven different magnetic properties were fabricated by uniting two nanoparticle types in varying weight ratios within individual SPs (Figure 8a, more information see Experimental). Each of the seven different magnetic marker types was integrated in the sealed seam of three pouch cells. The pouch cells were subsequently analyzed by MPS with the aim of distinguishing different SPs, while the three identical pouch cells per SP type should be identical. Indeed, it is found that different MPS spectra for pouch cells with different SP types are obtained. By changing the SP mass ratio starting with a nanoparticle mass ratio of 1:0 (Figure 8a, violet hue) and increasing the ratio to 0 : 1 (pale green hue), the MPS spectra decay faster, thus magnetically representing the used mass ratio (Figure 8b). Thus, most importantly, different battery pouch cells can be distinguished from another based on their MPS signal response. The three pouch cells per SP type are shown by three data sets of identical color. It is found that sufficient SNR with small error bars (standard deviation of each measurement) is obtained for up to the 13^th^ harmonic. However, slight signal deviations are found within the sets of three identical pouch cells. This may limit the unambiguous identification and distinction of different pouch cells. Repeated measurements confirm the robustness of MPS measurements (Figure S6, Supplementary Information). Observed signal variations can be most likely attributed to the manual fabrication of comparably small batch sizes of SPs and the manual integration into pouch cells. Thus, upon upscaling and automating these processes, we are confident that >10 magnetic codes will be distinguishable out of such pouch cells with the used materials. When other magnetic nanomaterials are used as well, the number of distinguishable codes might increase significantly, as discussed elsewhere.^[18]^ The exact number of distinguishable cell types will depend on the reproducibility of marker and cell fabrication in industrial scale, and magnetic readout with desired marker masses. With these identifiers, information such as cell chemistry, origin, or manufacturer could be obtained and adequate measures during the life cycle of a battery, including its sorting and recycling, could be conducted.

## Conclusions

The concept and practicality of utilizing magnetic SPs for pouch cell identification *via* MPS as an alternative to externally applied optical identifiers have been demonstrated. The investigation of three model scenarios revealed that the cell performance and detection of SPs are highly dependent on the integration location. The integration of magnetic SPs in the sealing seam of the pouch foil was found to be the most appropriate process. In addition to high detectability of the magnetic signal response, the magnetic additive is spatially separated from the electrochemically active material preventing unwanted effects on the performance of the cell. Integration in the electrodes addresses this crucial aspect with currently impracticable results. It is conceivable that the results of integration in the electrodes could be enhanced with an advanced SPs design and reduced marker quantity. In this work, we focused on the integration of SPs with different magnetic codes and the subsequent differentiation of pouch cells carrying them. It was demonstrated that multiple pouch cells can be distinguished based on their inherent magnetic SPs. The SPs could thus act as a complementary technology for currently employed tags such as QR‐codes with the advantage of being inside the battery cells rather than on their surface.

## Experimental Section

### Synthesis of Magnetic Identifiers

*Materials and reagents*: Iron(III) chloride hexahydrate (FeCl_3_ ⋅ 6H_2_O, >99 %), iron(II) chloride tetrahydrate (FeCl_2_ 4H_2_O, >99 %) and ammonia (NH_3_ (aq.), 25 %) were derived from *Carl Roth*. Polystyrene (PS, average Mw»192,000) was acquired from *Sigma Aldrich*. The utilized sodium dodecyl sulfate (SDS, ≥85 %) and was purchased from *Acros Organics*. Oleic acid (techn. 90 %) and Cobalt(II) chloride hexahydrate (CoCl_2_ ⋅ 6H_2_O, ≥98 %) were derived from *Thermo Scientific*. Chloroform (techn.) and all other commercially available chemicals were used without further purification. Deionized water was used for all synthesis procedures.

*Synthesis of SPIONsOA*: As described in previous works, iron oxide nanoparticles were generated *via* a precipitation reaction under basic conditions.[^18^, ^27^] To synthesize SPION1, an aqueous solution (deionized water, 125 mL) of FeCl_3_ ⋅ 6H_2_O (10.8 g, 40.0 mmol) and FeCl_2_ ⋅ 4H_2_O (3.98 g, 20.0 mmol) was prepared under stirring at room temperature (RT). The iron salt solution was combined with a solution of NH_4_OH (5 wt %, 180 mL) by pumping both solutions *via* a peristaltic pump (MCP from *Ismatec*, flow rate 500 mL ⋅ min^−1^) in a static mixer (plastic spiral bell mixer 7700924, *Nordson Deutschland GmbH*). The obtained dark precipitate, *i. e*. SPIONs, was stirred for approximately 60 s at RT. After magnetic separation with a permanent magnet (Neodym, N40) and purification with water (250 mL) three times, the SPIONs (4.5 g) were redispersed in water (500 mL). Oleic acid (6.41 g, 22.7 mmol) was added dropwise over 10 min and the reaction mixture was subsequently stirred for 45 min. After successful surface functionalization, the SPIONsOA were magnetically separated and subsequently washed with ethanol (techn., 200 mL) three times. The oleate functionalized SPIONs were redispersed in chloroform (techn., 50 mL) and sonicated for 1 min (*Branson* Ultrasonic Sonifier, output: 20, duty cycle: 50 %), yielding a stable ferrofluid with a gravimetrically determined solid content of about 7 wt %. For SPION2, FeCl_3_ ⋅ 6H_2_O (9.2 g, 40.0 mmol) and CoCl_2_ ⋅ 6H_2_O (1.43 g, 6.01 mmol) were used, while the amount of FeCl_2_ ⋅ 4H_2_O and the rest of the procedure was left unchanged.

*Synthesis of Nanocomposite Particles*: Nanocomposite particle (NCP) dispersions were prepared *via* a reaction‐free solvent evaporation method adapted from previous studies of S. Müssig.^[18]^ The synthesis procedure is exemplarily shown for nanocomposite particle dispersion NCPs1 with a ratio SPION1:PS of 4 : 1. The SP powder generated based on NCPs1 were used to evaluate the integration scenarios. A prepared stock solution of PS in chloroform (10 wt %, 2.01 g) was added to a dispersion of oleate functionalized SPIONs in chloroform (6.9 wt %, 11.67 g) in a 50 mL glass vial. Thereby, the total solid content was kept at mass of 1 g. Chloroform was added up to a total volume of 20 mL of the organic solvent. After addition of SDS solution (0.5 wt %, 20 mL), the reaction mixture was sonicated for 2 min (*Branson* Ultrasonic Sonifier, output: 20, duty cycle: 100 %) while cooling the mixture in an ice bath. The solvent was evaporated from the emulsified mixture under continuous stirring for 36 h at RT. The solid content was gravimetrically determined to 5.6 wt %. To demonstrate the distinguishability of different SP species, 2 nanocomposite particle dispersions NCP2 and NPC3 with a ratio SPIONx:PS 1:0 consisting of SPIONs1 and SPIONs2 respectively were generated by the same procedure without addition of polymer.

*Assembly of SPs via Spray‐drying*: The synthesized precursor NPC1 was spray‐dried to SPs using a Mini Spray Dryer B‐290 from *Büchi*. Therefore, the solid content was adjusted to 4 wt % with water. The assembly was performed using a two‐fluid nozzle and constant instrument parameters (inlet temperature: 85 °C, feed flow: 0.18 L⋅h^−1^, pressured gas flow: 414 L⋅h^−1^, volume flow: 32 m^3^⋅h^−1^, resulting in outlet temperature: 41 °C and vacuum: −76 mbar). For distinguishability demonstration, the 2 nanocomposite particle dispersions NPC2 and NPC3 containing SPIONs1 and SPIONs2 respectively were combined before spray‐drying so that the particles’ mass represented the desired ratios of 1:0, 2.7 : 1, 1.7 : 1, 1 : 1.7, 1 : 2.7, 1 : 5.7 and 0 : 1 within SPs.

### Manufacturing of Magnetically Marked Pouch Cells

*Fabrication of Pouch Cells with SPs located in the Cathode (Scenario 2)*: The manufacturing of water‐based NMC811 cathodes was conducted with 5 g of NMC811 (*Gelon*), a CMC binder of 0.2174 g (*Sigma Aldrich*) and 0.2174 g of conductive carbon Super C65 (*Imerys*) in 7.174 g of deionized water. The reference cathodes were produced without SPs, whereas the sample cathodes contain 10 wt % SPs (0.5435 g) in relation to the total powder mass used in the cathode manufacturing process. The water and binder mixture was stirred on a Vibramax at 750 rpm for approximately 14 h and further stirred with the added active material powders in a Speedmixer at up to 2400 rpm. Subsequently, the SPs were added into the slurry and mixed at 800 rpm for 2 min. Afterwards, the finished slurries were coated on 20 μm alumina foil *via* the doctor blading process with a velocity of 5 mm/s and a doctor blade gap of 225 μm and 270 μm for the reference and particle integrated cathodes, respectively, in order to achieve an area specific capacity of ~2.0 mAh/cm^2^. Subsequently, the samples were subjected to a drying process at 80 °C for 30 min prior to calendaring, which resulted in a porosity of 50 %. Following this, the cathodes were punched out in a rotary press to a size of 5 cm×3 cm. Finally, the cathodes were dried under vacuum of approximately 50 mbar at 110 °C for 12 h. Three full cells were built by using a graphite anode provided by *CustomCells* with an area specific capacity of 2.4 mAh/cm^2^ in a size of 5.2 cm×3.2 cm. The electrodes were separated by a Cellgrad 2500 separator and infiltrated with 540 μL of LP57+2 % VC (*Elyte*) electrolyte.

*Fabrication of pouch cells with SPs located in the anode (Scenario 2)*: Anodes were manufactured by mixing 0.375 g of the CMC binder (*Sigma Aldrich*) with 20.375 g of water overnight on a Vibramax at 750 rpm. Subsequently, 0.450 g of conductive carbon Super C65 (*Imerys*) and 13.8 g of graphite (*Timrex SLP50*) were added and stirred in a Speedmixer at up to 2400 rpm. Finally, the SBR emulsion (*Targray*) containing 0.375 g of SBR and 2.125 g of water was added and stirred at 800 rpm. For the anodes containing SPs, an additional step was performed, in which 10 wt % of magnetic SPs (1.380 g) were added and stirred at 800 rpm for 2 min. Subsequently, the slurries were coated on 20 μm copper foil at a velocity of 2.5 mm/s and a doctor blade gap of 205 μm and 215 μm for the reference and the particle containing anodes, respectively, to achieve an area specific loading of ~2.4 mAh/cm^2^. The anodes were calendared to a porosity of 40 % following pre‐drying at 80 °C for 30 min. The anodes were punched out to a size of 5.2 cm×3.2 cm and dried under vacuum of approximately 50 mbar at 110 °C for 12 h. Two different types of full cells were built. First, cells for electrochemical measurements using NCM622 cathodes provided by *CustomCells* with an area specific loading of 2.0 mAh/cm^2^. Second, three cells for MPS measurements with the self‐made reference cathodes, with the objective of obtaining an equivalent cell configuration in terms of layer thicknesses. The separator and electrolyte were the same as those described before.

*Fixation of SPs into the sealing seam and under the pouch foil (Scenario 1 and 3)*: To prepare two full cells each for scenarios 1 and 3, where the SPs are either fixed into the full cell under the pouch foil or integrated into the sealing seam, the same amount of particles as present in one final cathode or anode was used, representing 0.0160 g. In scenario 1, the amount of magnetic particles was scattered onto Kapton adhesive tape which was then fixed on the inside of one pouch foil. The prepared foil was always used on the cathode side of the final cells. To integrate the particles into the pouch foil, a sheet was folded and the particles were scattered into the fold. Once they were distributed the fold was sealed with a 6 mm wide seal bar at 175 °C under a pressure of approximately 200 N for 3 s. Subsequently, the requisite dimensions for constructing the full cells were punched out *via* a rotary press, while the folding edge was trimmed to create a sealing seam comparable to that of a conventional sealing procedure.

### Analytical Methods and Characterization

*Magnetic Particle Spectroscopy*: For MPS measurements, a complete pouch cell is placed on an MPS sensor (*Fraunhofer IIS*). A sinusoidal alternating magnetic field of 20 kHz was applied in a magnetic field range of ±200 Oe. The shown MPS spectra were background corrected with reference spectra of pouch cells without magnetic markers and received by averaging 21 measurements, whereas the individual measurement time was 1 s. To analyze the raw SP powder, MPS measurements were performed on approximately 3 mg of the powdered sample using an MPS volume sensor (*Pure Devices GmbH*) in an alternating field of 20 kHz from −300 to +300 Oe. The spectrum represents an average of ten individual measurements at 37 °C.

*Scanning Electron Microscopy*: SEM was performed on SP powder samples adhered on carbon pads using a JSM−F100 (*JEOL*) with an acceleration voltage of 3 kV. The SP‐based samples were sputtered with Pt before SEM analysis with a 108SE (*Cressington*). SEM of the electrodes was performed at cross‐section images prepared by argon ion milling with a *JEOL* SM‐0910 cross section polisher. The images were recorded with an acceleration voltage of 2 kV at a working distance of 3.5 mm with a secondary ion detector using a ZEISS Ultra 55 (*Carl Zeiss AG*).

*Superconducting quantum interference device*: Magnetization curve measurements were conducted in a magnetic field range of ±30 000 Oe using a MPMS3 (*Quantum Design Inc*.). At 300 K, the measurement speed was set to 5 Oe s^−1^ between ±5000 Oe and 50 Oe s^−1^ beyond.

*Laser light diffraction*: Magnetization curve measurements were conducted in a magnetic field range of ±30 000 Oe using a MPMS3 (*Quantum Design Inc*.). At 300 K, the measurement speed was set to 5 Oe s^−1^ between ±5000 Oe and 50 Oe s^−1^ beyond.

*Galvanostatic cycling including differential capacity analysis*: Cycling tests were performed on three cells for each configuration using a Maccor Series 4000 electrochemical workstation (*Maccor Inc*.) in temperature chambers maintained at 25 °C (IPP260, *Memmert GmbH+Co. KG*). After a rest period of 24 h, all cells were subjected to five formation cycles at 0.1 C and a voltage range of 3.0 to 4.2 V. The full cells with SPs integrated into the cathode and the corresponding references were then cycled for 100 cycles at 0.5 C in a voltage range of 2.7 to 4.2 V. Two control cycles at 0.1 C were performed after 50 cycles. The full cells with SPs integrated in the anode and the corresponding references were subjected to 500 cycles at 0.1 C in the same voltage range, with control cycles every 125 cycles. The cells were always charged with a constant‐current constant‐voltage method and discharged with a constant‐current step. The termination condition of the constant voltage step was set at a current of 0.05 C.

*Impedance*: Electrochemical impedance measurements were performed using a VMP300 galvanostat‐potentiostat (*BioLogic GmbH*) on one cell for each configuration. Before measuring the impedance spectra, the cells underwent five formation cycles at 0.1 C. To ensure an equilibrium state of the cells, a constant voltage step of 3 h was performed prior to the measurements. The impedance spectra were obtained successively at 3.8 V with an alternating current (AC) voltage of 5 mV amplitude over the frequency range from 10 mHz to 6 MHz.

*X‐ray diffractometry*: The electrodes were measured using a SmartLab diffractometer (*Rigaku Corporation*) in the range of 2Θ=10–80°, at a scan rate of 0.3°/min and a step size of 0.01°. The cycled electrodes were washed in dimethyl carbonate to remove electrolyte salt residues and dried under vacuum prior to the measurement.

## Conflict of Interests

The authors declare no conflict of interest.

1

## Supporting information

As a service to our authors and readers, this journal provides supporting information supplied by the authors. Such materials are peer reviewed and may be re‐organized for online delivery, but are not copy‐edited or typeset. Technical support issues arising from supporting information (other than missing files) should be addressed to the authors.

Supporting Information

## Data Availability

The data that support the findings of this study are available from the corresponding author upon reasonable request.
