# Supplementary material for: Magnetic Supraparticles as Identifiers in Single‐Layer Lithium‐Ion Battery Pouch Cells
Source: ChemSusChem. 2024 Nov 10;18(6):e202401142. doi: 10.1002/cssc.202401142 (PMC11912094; doi:10.1002/cssc.202401142)
Supplement: Supplementary file 1 — Supporting Information [file CSSC-18-e202401142-s001.pdf]

ChemSusChem

Supporting Information

Magnetic Supraparticles as Identifiers in Single-Layer Lithium-Ion Battery Pouch Cells

Sara Li Deuso, Simon Ziegler, Daniel Weber, Felix Breuer, Daniel Haddad, Stephan Müssig, Andreas Flegler, Guinevere A. Giffin,* and Karl Mandel*

Supplementary Information

Magnetic Supraparticles as Identifiers in Single-Layer Lithium-Ion Battery Pouch Cells

Sara Li Deuso [†a], Simon Ziegler [†b], Daniel Weber [c], Felix Breuer [c], Daniel Haddad [c], Stephan Müssig [a], Andreas Flegler [b], Guinevere A. Giffin*[b] and Karl Mandel*[a,b]

[†] These authors contributed equally to this work.

[a] Department of Chemistry and Pharmacy, Inorganic Chemistry, Friedrich-Alexander-Universität Erlangen-Nürnberg (FAU), Egerlandstraße 1, D-91058 Erlangen

[b] Fraunhofer R&D Center Electromobility, Fraunhofer Insitute for Silicate Research (ISC), Neunerplatz 2, D-97082 Würzburg

[c] Magnetic Resonance and X-Ray Department (MRB), Development Center X-ray Technology (EZRT), Fraunhofer Institute for Integrated Circuits (IIS), Am Hubland, D-97074 Würzburg

*Corresponding authors:

E-mail: guinevere.giffin@isc.fraunhofer.de (G. A. Giffin); karl.mandel@fau.de (K. Mandel)

Supplementary Results

Magnetic particle spectroscopy (MPS) is a sensitive technique that is dependent on the magnetizable surrounding, which could influence the field at the measurement location. Metallic materials are one example of a delicate influence on this. Consequently, the spectrum of raw SP powder may be altered to that of the particles inside a battery environment. The objective of the MPS measurement is to quantify the non-linear magnetization curve (see Figure 3a) and subsequently calculate the MPS spectrum (the "code") of the SPs. In the case of surrounding metallic materials, such as pouch foil, copper foil of the anode, aluminum foil of the cathode or metallic components of the battery materials used, these can influence the excitation field at the location of the SP (due to eddy currents, shielding effects, field focusing, etc.). Consequently, this can also affect the MPS spectrum. Furthermore, an MPS surface sensor has a field distribution. When used on pouch cells, the SPs are detected by this field distribution resulting in a superposition of numerous MPS spectra. If the field distribution at the locations of the SPs undergoes a change due to the context of the SPs (e.g. various metallic foils with specific geometry), this will influence the MPS spectrum that is effectively measured.

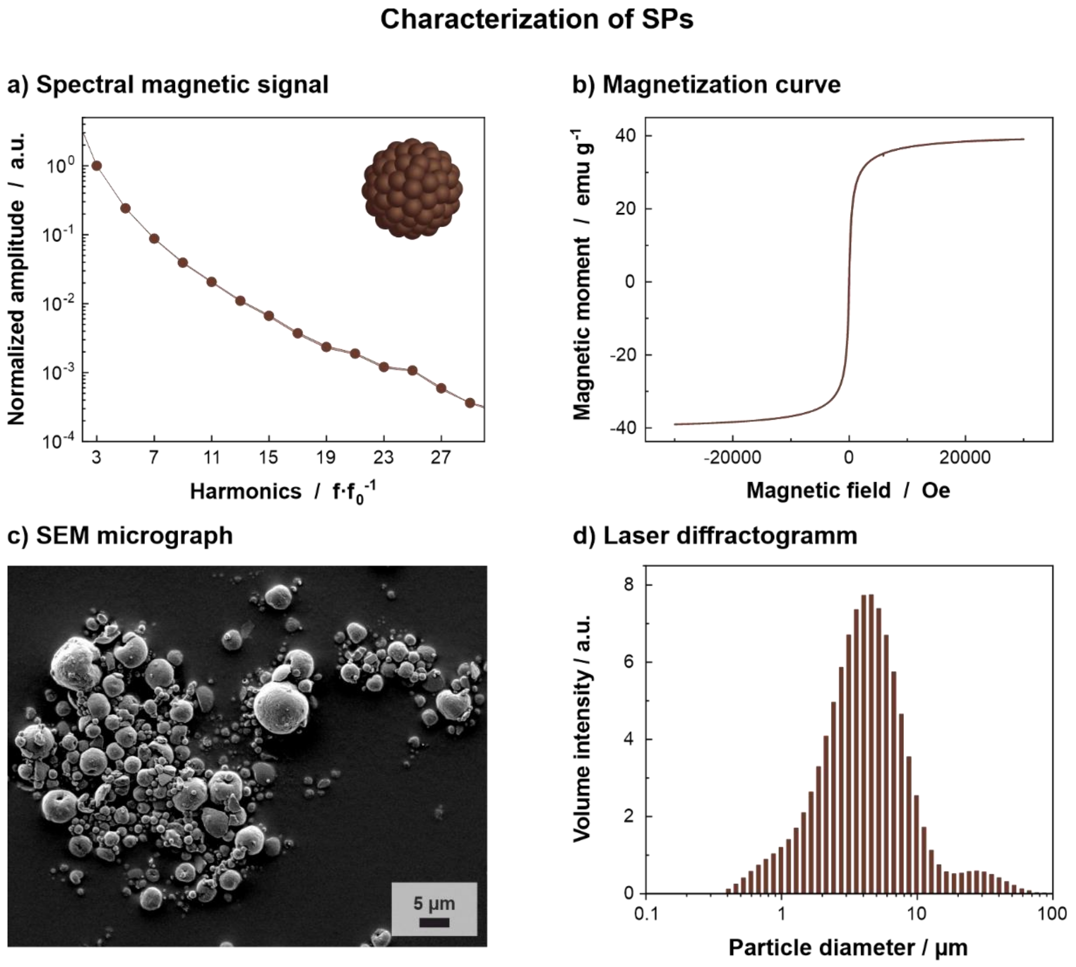

Figure S1. Magnetic characterization of SPs including MPS spectrum of raw SP powder measured with a MPS unit together with a MPS volume sensor from *Pure Devices GmbH* (a) and a magnetization curve measured with a SQUID (b). Size distribution of the magnetic SPs is visualized in a SEM micrograph overview (c) and was measured with laser light diffraction (d).

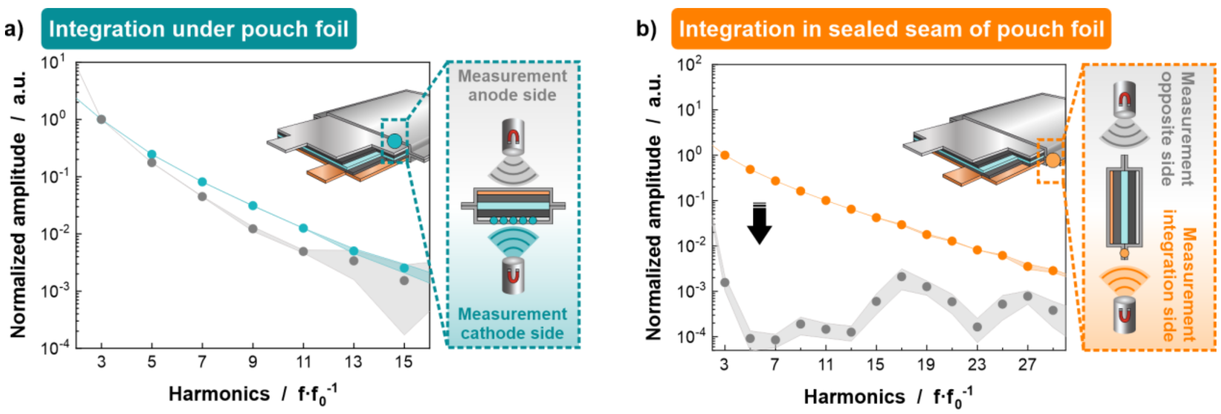

Figure S2. MPS measurements of a second, identically prepared pouch cell exhibits similar signal trends for integration a) under the pouch foil and b) the sealing seam of the pouch foil.

Impedance spectroscopy of marked vs. reference cells

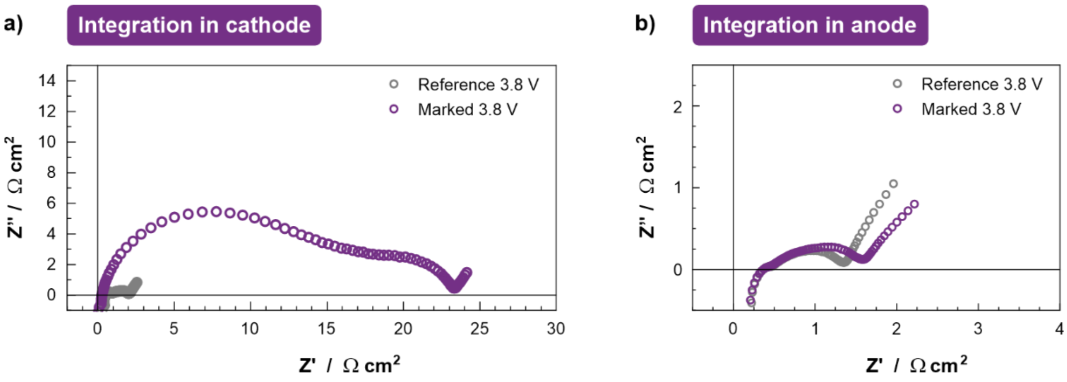

Figure S3. Impedance measurements at 3.8 V of cells with integrated particles and their corresponding references for (a) SPs integrated in the cathode and (b) SPs integrated in the anode.

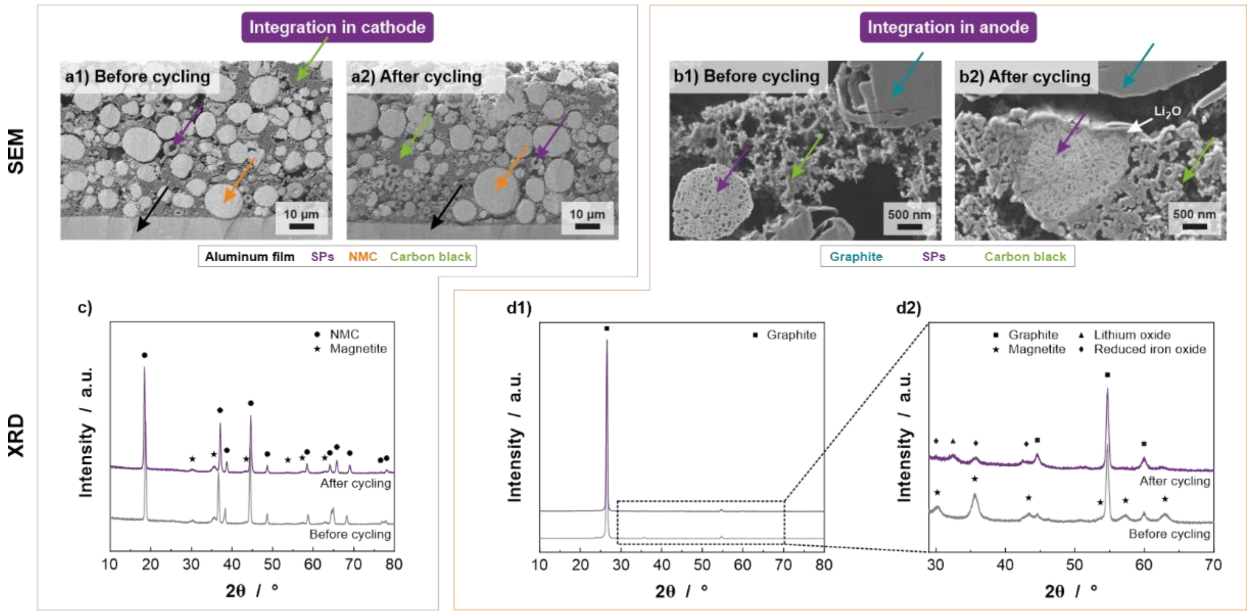

Figure S4. Characterization of the electrodes was conducted by SEM analysis of (a) a pristine cathode, and (b1) and (b2) a pristine and a cycled anode, respectively. The materials present in the electrodes are carbon black (green) and SPs (violet) for both electrodes and NMC (orange) and graphite (turquoise) for the cathode and anode,

respectively. X-ray diffractograms of cathodes (c) and anodes (d1 and d2) before and after cycling are displayed with the assignment of the present reflections to NMC (ICSD#40260681, dots) and magnetite (ICSD#40210451, stars) for the cathode and graphite (ICSD#411487, squares), magnetite (stars), lithium oxide (ICSD#530334, triangle) and reduced iron oxide (ICSD#247035, rue) for the cycled anode.

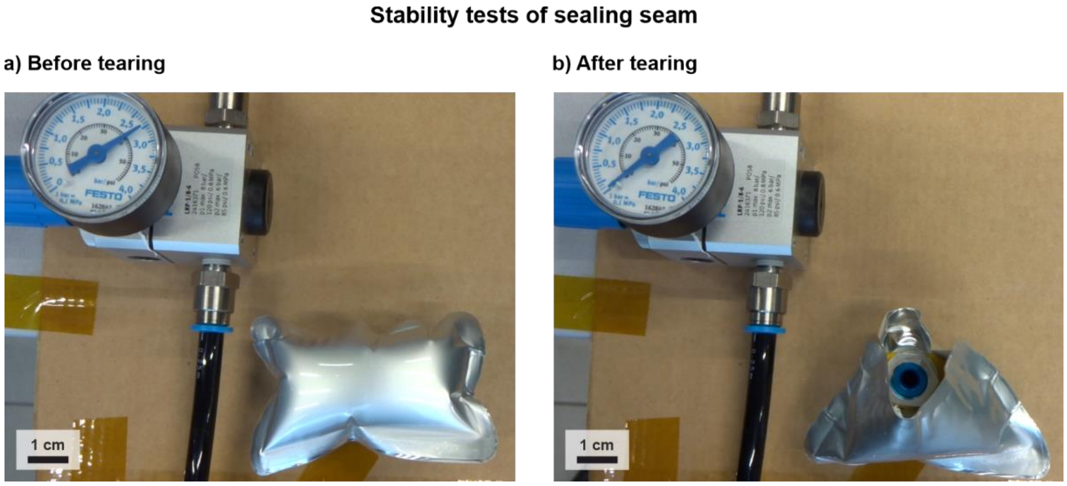

Figure S5. Images of a stability test of a pouch cell by inflating the empty sample cell until failure of a sealing seam and measuring the achieved maximum pressure as well as identifying the failure location via a precision manometer by video recording.

To investigate the robustness of our measurement procedure we conducted several experiments: firstly, we measured each of two selected pouch cells with different magnetic codes for an extended measurement period of 30 minutes (Fig. S6a). The obtained result demonstrates that the measuring apparatus gives accurate results for both pouch cells with no or minimal signal drift and fluctuations. Both codes can reliably be distinguished from each other.

Secondly, we manually positioned three pouch cells with the same code for two similar codes with multiple alternated positioning and conducted measurements. Here, slight differences in the signal response were obtained, making the separation of the codes more difficult and potentially less reliable (Fig S6b). This can be most likely attributed to the fact that the codes as well as the pouch cells are currently manually fabricated, thus potentially offering inhomogeneity. However, we expect that a more standardized automated fabrication process will allow us to further improve the accuracy with improved code separation ability. Thus, thirdly, we investigated the influence of the manual positioning process. To this end we alternated the measurement of two pouch cells with different magnetic codes 20 times. The obtained results indicate that if one can ensure a reproducible setup for the positioning of the pouch cells, the repeated measurement of the same pouch cell delivers a very reliable measure of the marker code, still allowing for robust identification of the codes.

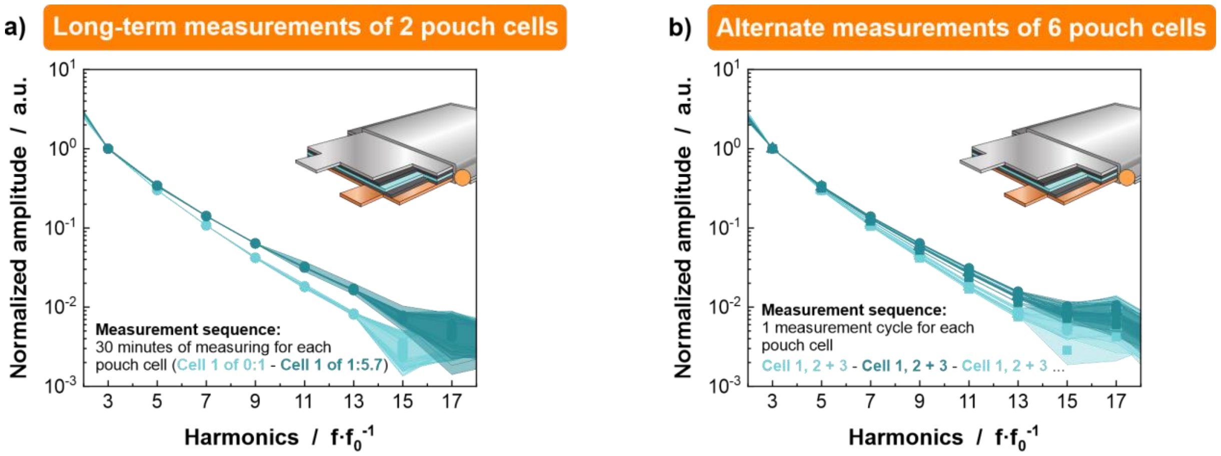

Figure S6: a) MPS measurement of two pouch cells with identical magnetic code (ratio 0:1, light blue) and two pouch cells with different magnetic code (ratio 1:5.7, dark blue) for an extended measurement period of 30 minutes per cell. b) Two sets of pouch cells, each containing three cells with identical magnetic SP types, were alternately placed on the MPS sensor 20 times.
